# Platelet Derived Growth Factor-AA Correlates With Muscle Function Tests and Quantitative Muscle Magnetic Resonance in Dystrophinopathies

**DOI:** 10.3389/fneur.2021.659922

**Published:** 2021-06-11

**Authors:** Alicia Alonso-Jiménez, Esther Fernández-Simón, Daniel Natera-de Benito, Carlos Ortez, Carme García, Elena Montiel, Izaskun Belmonte, Irene Pedrosa, Sonia Segovia, Patricia Piñol-Jurado, Ana Carrasco-Rozas, Xavier Suárez-Calvet, Cecilia Jimenez-Mallebrera, Andrés Nascimento, Jaume Llauger, Claudia Nuñez-Peralta, Paula Montesinos, Jorge Alonso-Pérez, Eduard Gallardo, Isabel Illa, Jordi Díaz-Manera

**Affiliations:** ^1^Neuromuscular Disorders Unit, Neurology Department, Hospital de la Santa Creu i Sant Pau, Departament de Medicina. Universitat Autònoma de Barcelona, Barcelona, Spain; ^2^Neurology Department, Neuromuscular Reference Center, University Hospital of Antwerp, Antwerp, Belgium; ^3^Biomedical Network Research Centre on Rare Diseases (CIBERER), Barcelona, Spain; ^4^John Walton Muscular Dystrophy Research Centre, International Centre for Life, Newcastle University, Newcastle upon Tyne, United Kingdom; ^5^Neuromuscular Unit, Neuropediatrics Department, Institut de Recerca Sant Joan de Déu, Hospital Sant Joan de Déu, Barcelona, Spain; ^6^Rehabilitation and Physiotherapy Department, Hospital de la Santa Creu i Sant Pau, Universitat Autònoma de Barcelona, Barcelona, Spain; ^7^Departamento de Genética, Microbiología y Estadística, Universidad de Barcelona, Barcelona, Spain; ^8^Radiology Department, Hospital de la Santa Creu i Sant Pau, Universitat Autònoma de Barcelona, Barcelona, Spain; ^9^Philips Healthcare Iberia, Madrid, Spain

**Keywords:** PDGF, MRI, biomarker, Duchenne, Becker, dystrophinopathy

## Abstract

**Introduction:** Duchenne (DMD) and Becker (BMD) muscular dystrophy are X-linked muscular disorders produced by mutations in the DMD gene which encodes the protein dystrophin. Both diseases are characterized by progressive involvement of skeletal, cardiac, and respiratory muscles. As new treatment strategies become available, reliable biomarkers and outcome measures that can monitor disease progression are needed for clinical trials.

**Methods:** We collected clinical and functional data and blood samples from 19 DMD patients, 13 BMD patients, and 66 healthy controls (8 pediatric and 58 adult controls), and blood samples from 15 patients with dysferlinopathy (DYSF) and studied the serum concentration of 4 growth factors involved in the process of muscle fibrosis. We correlated the serum concentration of these growth factors with several muscle function tests, spirometry results and fat fraction identified by quantitative Dixon muscle MRI.

**Results:** We found significant differences in the serum concentration of Platelet Derived Growth Factor-AA (PDGF-AA) between DMD patients and pediatric controls, in Connective Tissue Growth Factor (CTGF) between BMD patients and adult controls, and in and Transforming Growth Factor- β1 (TGF-β1) between BMD and DYSF patients. PDGF-AA showed a good correlation with several muscle function tests for both DMD and BMD patients and with thigh fat fraction in BMD patients. Moreover, PDGF-AA levels were increased in muscle biopsies of patients with DMD and BMD as was demonstrated by immunohistochemistry and Real-Time PCR studies.

**Conclusion:** Our study suggests that PDGF-AA should be further investigated in a larger cohort of DMD and BMD patients because it might be a good biomarker candidate to monitor the progression of these diseases.

## Introduction

Duchenne (DMD) and Becker (BMD) muscular dystrophy are X-linked recessive disorders produced by mutations in the *DMD* gene which encodes the protein dystrophin (1). Dystrophin is a cytoplasmic protein which links the intracellular cytoskeleton network to transmembrane components of the dystrophin-glycoprotein complex, including dystroglycan, sarcoglycans, and sarcospan (2). Its role is to stabilize the membrane during muscle contraction to prevent contraction-induced damage (3).

DMD has an homogeneous clinical picture characterized by early onset of muscle weakness progressing during childhood and leading to loss of ambulation during adolescence (4). All DMD patients invariably develop cardiac and respiratory involvement, which are the main causes of death. In contrast, BMD is an heterogeneous disease characterized by muscle weakness of variable severity that can start during childhood but also late in adulthood (5). BMD patients can also develop respiratory and cardiac muscle involvement. Despite having substantial phenotypic differences, DMD and BMD share common pathological pathways. Mutations in the *DMD* gene lead to abnormal dystrophin expression which results in a fragile muscle membrane susceptible to be damaged during contraction (3, 6). Continuous muscle damage leads to persistent inflammation, muscle fiber necrosis and replacement by fat and fibrous tissue, producing permanent weakness and disability (6).

In slow progressive diseases, the identification of functional changes over a short period is not an easy task. In recent years several outcome measures such as the 6 Minutes-Walk Test (6MWT), the Timed Up-and-Go Test (TuGo) or the North Star Ambulatory Assessment (NSAA) have been developed to monitor muscle function in natural history studies and clinical trials (7–10). Additionally, specific tests have been developed to assess upper limb muscle function such as the Brooke Upper Extremity Functional Rating (Brooke scale) or the Performance of the Upper Limb (PUL) (11–13). However, as muscle fiber necrosis and replacement by fat is a slowly progressing process, changes in motor functional tests could take time to be evident. In view of this limitation, muscle magnetic resonance imaging (MRI) and serum biomarkers have been investigated as potential outcome measures that might complement muscle function tests (14–16). Although muscle MRI is becoming more widely accepted as a tool to monitor patients, there is not a serum biomarker accepted so far that correlates with several muscle function tests and that could therefore be considered useful for clinical trials or natural history studies.

The main aim of this study was to analyze the serum concentration of a group of growth factors related to the process of muscle fibrosis in a cohort of 19 patients with DMD and 13 patients with BMD and study whether there was a correlation with the results of muscle function tests and quantitative MRI (qMRI).

## Materials and Methods

### Study Design and Participants

We report the results of a transversal study of a cohort of 19 DMD and 13 BMD patients seen at Hospital de la Santa Creu i Sant Pau (HSCSP) and Hospital Sant Joan de Déu (HSJD) in Barcelona. All patients included in the study were assessed using muscle function tests and spirometry and filled out a daily life activities questionnaire. qMRI was not a mandatory test and was performed in a subset of patients followed-up in HSCSP only. Blood samples were obtained before the muscle function assessment. The HSCSP and HSJD Ethics Committees approved the study, and all participants signed an informed consent form. All study procedures were performed in accordance with Spanish regulations.

Inclusion criteria for the study were: (1) Diagnosis of DMD or BMD confirmed by the identification of pathogenic variants in the *DMD* gene and (2) willingness to complete muscle function tests, respiratory assessment, and daily life activities scale.

### Functional Assessment

Muscle function tests were performed by physiotherapists with considerable experience in neuromuscular disorders. We assessed patients using timed tests, functional ability scales, and measurement of muscle strength. Timed tests included the 6MWT, the 10 Meter Run/Walk test (10MWT), the Time to Climb Up (Tup4) and Down (Tdo4) Four Steps and the Time to Rise from the Floor (TRF). Functional ability included the NSAA and the Motor Function Measure 20-item scale (MFM-20) (17). The MFM-20 was also analyzed based on its 3 dimensions: D1 (standing and transfers), D2 (axial and proximal), and D3 (distal). PUL test was used to assess upper extremity function in both ambulant and non-ambulant individuals. Egen Klassification was used to assess mobility in wheelchair-bound patients (18). Muscle strength was studied using the Medical Research Council scale (MRC). For analysis, 5-point MRC power grades were converted to an 11-point scale as previously described (19). We assessed bilateral shoulder flexion, shoulder abduction, elbow flexion, elbow flexion brachioradialis, wrist extension, wrist flexion, hip adduction, hip abduction, hip flexion, hip extension, knee extension, knee flexion, ankle eversion, ankle inversion, ankle plantar flexion, and ankle plantar extension. Grip and pinch strength were studied using a hand dynamometer and the best of 3 attempts was used for analysis. Daily life activities were assessed using the ACTIVity LIMitations scale for patients with upper and/or lower limb impairments (ACTIVLIM) (20). We also assessed respiratory function using the Carefusion Microlab ML 3500 MK8 spirometer (Carefusion, Yorba Linda, CA, USA). We analyzed the Forced Vital Capacity (FVC) while seating and lying and the Forced Expiratory Volume in the first second (FEV1).

### Quantification of Growth Factors in Serum Samples

We measured the serum concentration of the following growth factors related with muscle fibrosis: Serum Platelet-Derived Growth Factor BB (PDGF-BB), Transforming Growth Factor β1 (TGF-β1), Platelet-Derived Growth Factor AA (PDGF-AA) and connective tissue growth factor (CTGF). Blood was centrifuged at 1,600 g for 9 min at 4°C. Samples were aliquoted and stored at −80°C until analysis. PDGF-BB and TGF-β1 levels were measured using commercial enzyme-linked immunosorbent assay (ELISA) kits (R&D, Minneapolis, MN, USA), according to the manufacturer's instructions. PDGF-AA human ELISA kit was provided by ThermoFisher (Thermo Fisher Scientific, Nepean, Canada) and CTGF by EIAAB Science Co (Wuhan, China). Minimum detectable cytokine concentrations for these assays were measured to be 1.7 pg/ml for TGF-β1, 15 pg/ml for PDGF-BB, 40 pg/ml for PDGF-AA and 0.18 ng/ml for CTGF. Samples were measured in duplicate and read on a microplate reader Beckman Coulter AD 340 (Beckam-Coulter, Brea, CA, USA) with AD-LD software.

These growth factors were determined in the 19 DMD patients, 13 BMD patients, 15 patients with mutations in the *DYSF* gene that were being visited in HSCSP (from now on DYSF patients) and in 66 healthy controls. The healthy controls were divided into a pediatric group (<15 years old) and adult group (≥15 years old). Since the average age of DMD cohort is significantly different from the DMB and DYSF cohort, the DMD group was compared to the pediatric control group while the DMB and DYSF group was compared to the adult control group.

### Muscle MRI

7 DMD and 8 BMD patients underwent thigh muscle MRI in a 1.5T Ingenia MR system (Philips Healthcare, Best, the Netherlands) at HSCSP. Axial 3D FFE Dixon sequence was acquired with the following parameters: TR/TE = 5.78/1.84 ms, flip angle = 15°, voxel size = 1 × 1 × 3 mm and FOV 520 × 340 × 300 mm. Water and fat images were automatically obtained from the Dixon acquisition using a single peak model. One investigator (A.A-J) estimated fat content in the images using the PRIDE (Philips Research Image Development Environment) tool developed for this purpose. This tool provides the fat fraction (FF), defined as fat/(fat+water). Regions of interest (ROIs) were manually drawn on five slices on the right thigh of the following muscles: *rectus femoris, vastus intermedius, vastus lateralis, vastus medialis, adductor magnus, sartorius, gracilis, biceps femoris long head, semitendinosus, and semimembranosus*. We intended to include slices of the proximal, middle and distal part of the thigh. To do so, we identified the head of the femur and set the first slice at 5 centimeters below the head. The remaining four slices were distally distributed in the thighs with a separation of 20 slices between them as previously published (21). In patients in which individual muscles in the posterior or anterior compartment of the thigh could not be analyzed due to a complete fat replacement, a ROI was manually drawn over the whole compartment. Neurovascular bundles were avoided. An average of the fat fraction of the 5 slices was calculated for each muscle, and weighted averages were calculated by normalizing the FF by the area of the muscle (bigger muscles had higher weight) to obtain the global thigh FF.

### RNA Extraction and Reverse Transcription

Total RNA was extracted from triceps or biceps biopsy samples from 2 healthy controls and 2 DMD patients using RNeasy® Micro Kit (Qiagen, Hilden, Germany) following manufacturer's instruction. RNA was quantified using a nanodrop ND-1000 spectrophotometer (Nanodrop Technologies Inc., Wil- mington, DE, USA). One μg of total RNA was reverse-transcribed to complementary DNA (cDNA) using the High Capacity cDNA Reverse Transcription Kit (Applied Biosystems, Foster City, CA, USA).

### Real-Time Quantitative PCR Analysis

Real-Time PCR (qPCR) was performed using the TaqMan® Universal PCR Master Mix (Applied Biosystems, Foster City, CA, USA) and a 7900HT Fast Real-Time PCR System (Applied Biosystems, Foster City, CA). All mRNA- specific FAM-labeled primers/probe were purchased from Applied Biosystems. Relative quantification was performed using the comparative Ct method and all results were compared with the control samples. GAPDH was used as endogenous control.

### Immunohistochemistry

PDGF-AA immunohistochemistry was performed from paraffinized muscle. The samples were deparaffinized (xylol, absolute ethanol, 95° ethanol, and 70° ethanol), placed in distilled water and pretreated with 10 mM citrate (pH 6) at 100°C. Then samples were washed in PBS and incubated in blocking solution (4% bovine serum albumin). The primary anti-PDGF-AA antibody (Merck Millipore, Darmstadt, Germany) was then added at room temperature for 1 h. After 3 washes, it was incubated with biotinylated anti-rabbit antibody (Vector Laboratory Inc. Burlingame, CA) for 1 h at room temperature and then avidin-biotin peroxidase complex (Dako, Glostrup, Denmark) was added. Finally, the sections were soaked in Mayer's hematoxylin for 10 s, washed under running water and mounted in the aqueous mounting medium Aquatex (Merck Millipore, Darmstadt, Germany).

### Data Analysis

Non-parametric tests were used for the statistical analysis as we demonstrated that the distribution of the variables was not uniform as studied using Kolmogorov-Smirnov and did not follow a normal distribution as studied using Shapiro-Willis test. The Mann-Whitney U test was used to identify significant differences in variables between 2 groups. Non-parametric Kruskal–Wallis test analysis was used to assess differences among 3 groups followed by Dunn's multiple comparison post-test. We used Spearman's rank correlation (coefficient reported as ρ) to investigate any correlation between the serum concentration of growth factors and the results of the muscle function tests, quality of life scales and the thigh FF obtained using Dixon imaging. Correlation coefficients higher than 0.6 were considered good. Because the objective of our research was to explore the possibilities of these growth factors for further studies, we decided not to use multiple comparisons. The results of all statistical studies were considered significant if *P*-value was lower than 0.05. Statistical studies were performed using IBM SPSS® Statistics software version 21. Graphs were created using Graphpad software and R software.

## Results

### Cohort Description

A total of 19 patients with DMD (mean age 10 ± 5.6 years), 13 patients with BMD (mean age 31 ± 20.6 years), 15 DYSF patients (mean age 46 ± 18.6 years), 8 pediatric controls (mean age 10 ± 2 years) and 58 adult controls (mean age 43 ± 15.32 years) were enrolled in the study. There was no statistical difference in age between the DMD group and the pediatric controls (Mann-Whitney *U*-test, *p* = 0.979) and among the BMD, DYSF group and the adult control group (Kruskall-Wallis test, *p* = 0.052) We identified deletions in the *DMD* gene in 71.9% of patients. Duplications accounted for 12.5% of cases and 15.6% were due to point mutations. Among the DMD patients, 13 were ambulant (68.42%) and 6 non-ambulant (31.58%). In the BMD group, 8 patients were ambulant (61.5%) and 5 non-ambulant (38.5%). Five DYSF patients were non-ambulant (33.33%). Table 1 describes the mean demographic and clinical data of all patients included in the cohort.

**Table 1:** Clinical features of the subjects included in the study.

	**Patients**				
	**DMD**	**BMD**	**DYSF**	**Pediatric controls**	**Adult controls**
Number of patients	19	13	15	8	58
Age (years)	10 ± 5.6	31 ± 20.6	46 ± 18.6	10 ± 2	43 ± 15.32
Non-ambulant	6 (31.58%)	5 (38.5%)	10 (66.7%)	0	0
Ventilator support	1 (5.3%)	2 (15.38%)	1 (6.66%)	0	0
Corticoid treatment	15 (78.94%)	0	0	0	0
qMRI	7 (36.8%)	8 (61.5%)	0	0	0

*Age is given as mean ± standard deviation. Frequencies are given as absolute number followed by the percentage in brackets. DMD, Duchenne Muscular Dystrophy; BMD, Becker muscular dystrophy; DYSF, dysferlinopathy; qMRI, quantitative muscle magnetic resonance imaging*.

### Growth Factor Serum Levels Comparison Between DMD, BMD, and Age Matched Controls

**Figure 1:**
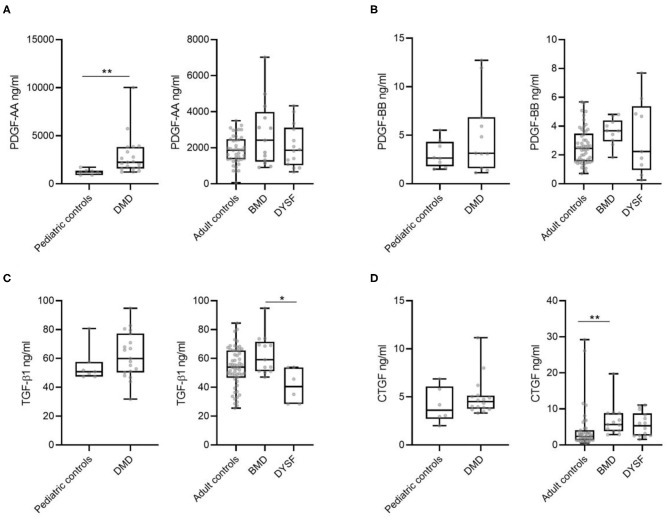
Serum levels of different growth factors in DMD, BMD, DYSF and in pediatric and adult healthy controls. **(A)** PDGF-AA, **(B)** PDGF-BB, **(C)** TGF-β1, and **(D)** CTGF levels were measured. Variables are represented as median and interquartile range (IQR). Statistical significance of the results by Mann-Whitney test for the DMD compared against pediatric controls and Kruskal-Wallis test/Dunn's multiple comparison analysis for the BMD, DYSF, and adult controls: *p ≤ 0.05 and **p ≤ 0.01.

Our first aim was to study whether there were differences in growth factor serum levels between DMD, BMD and DYSF patients compared to controls. We compared serum levels of PDGF-AA, PDGF-BB, CTGF, and TGF-β1 between DMD and pediatric healthy controls whereas BMD and DYSF patients were compared against adult healthy controls (Figure 1). In the case of DMD patients, we only observed significant differences in the serum levels of PDGF-AA as shown in Figure 1A. The median and IQR of PDGF-AA serum levels were 2245.32 ng/ml (IQR: 1589.94-3832.68) in DMD patients compared to 1242.45 ng/ml (IQR: 915.05-1373.13) in pediatric controls (Mann-Whitney *U*-test, *p* = 0.002). In the case of BMD and DYSF patients we observed significant differences in the serum levels of CTGF between BMD and adult controls and of TGF-β1 between BMD and DYSF patients as shown in Figures 1C,D. The median and IQR of CTGF serum levels were 5.66 ng/ml (IQR: 3.82-8.82) in BMD patients compared to 2.37 ng/ml (IQR: 1.35-4.07) in adult controls (Kruskal-Wallis test/Dunn's multiple comparison analysis, *p* = 0.004). The median and IQR of TGF-β1 serum levels were 59.10 ng/ml (IQR: 51.11-71.51) in BMD patients compared to 40.42 ng/ml (IQR: 28.44-53.75) in DYSF patients (Kruskal-Wallis test/Dunn's multiple comparison analysis, *p* = 0.01). Although PDGF-AA did not show statistical differences, there was a tendency toward increased levels in BMB patients 2415.60 ng/ml (IQR: 1228.59-3981.10) compared to adult controls 1859.62 (IQR: 1363.08-2477.52) (Kruskal-Wallis test/Dunn's multiple comparison analysis, *p* = 0.06).

### Correlation of Growth Factor Serum Samples With Muscle Function Test Results

**Figure 2:**
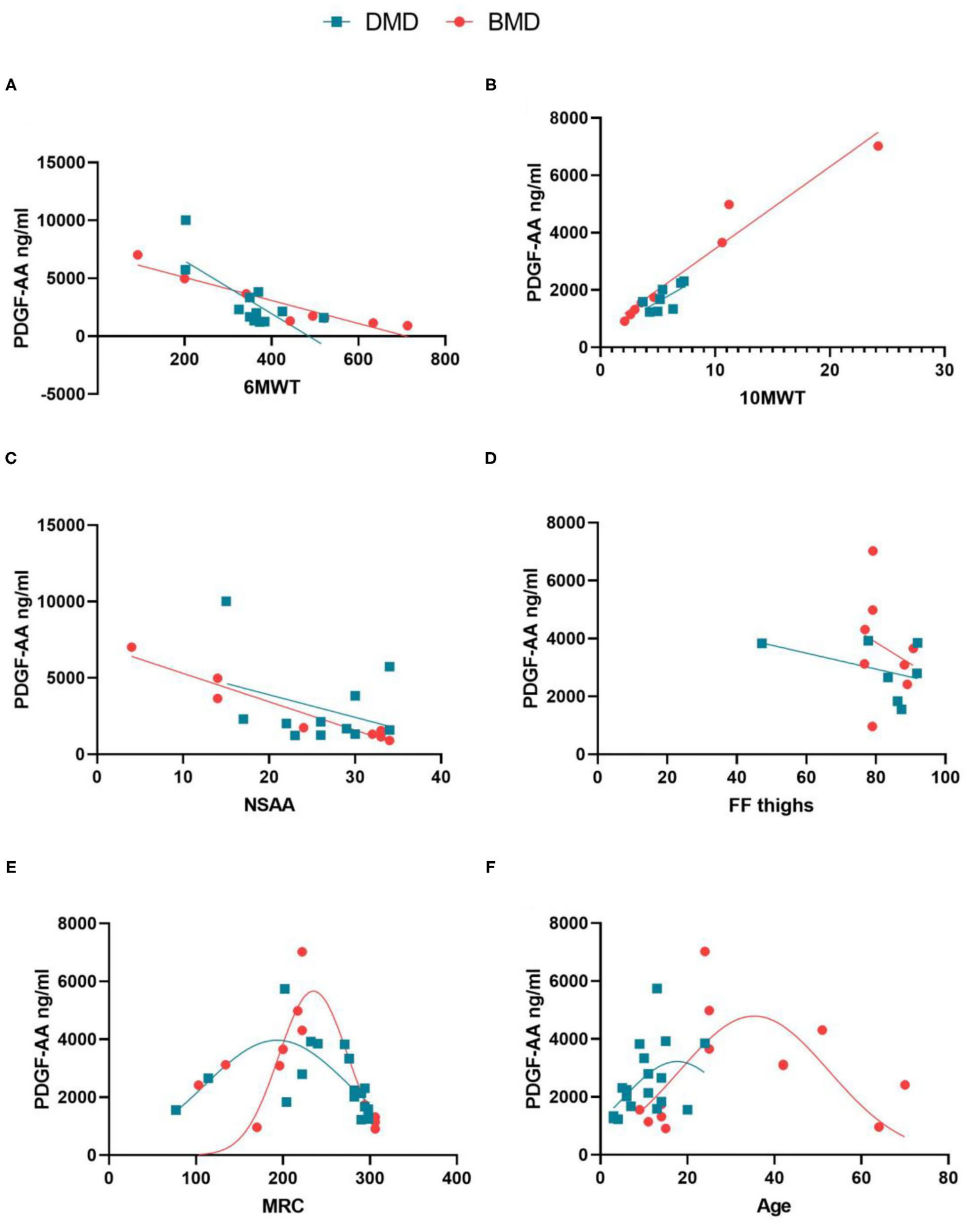
Correlation between PDGF-AA levels and different muscle tests in patients with DMD and BMD. **(A)** Correlation between PDGF-AA and the results of the 6MWT; **(B)** Correlation between PDGF-AA 10 MWT; **(C)** Correlation between PDGF-AA and results of the NSAA scale; **(D)** Correlation between PDGF-AA and thigh fat fraction; **(E)** Distribution of PDGF-AA serum levels depending on the muscle strength quantified using MRC score; **(F)** distribution of PDGF-AA serum levels depending on the age of the patients. Spearman test was performed, R, Correlation coefficient; p, statistical significance. Red lines: BMD patients; blue lines: DMD patients.

In a second step, we analyzed if there was a correlation between growth factor serum concentration and the results of muscle function tests, spirometry, daily life activities scales and Dixon MRI in DMD and BMD patients. The growth factor that showed a larger number of significant correlations was PDGF-AA as it is shown in Table 2. In the case of DMD, PDGF-AA serum levels correlated with the results of the 10MWT, the 6MWT, the MRC scale. and the dimension 3 of the MFM scale. In the case of BMD, PDGF-AA correlated with the results of the 10MWT, the 6MWT, the Tup4, Tdo4, TRF and the NSAA, FVC sitting, FEV1 and the thigh FF. Figure 2 shows graphs plotting these correlations. Moreover, we analyzed the distribution of PDGF-AA values in relation to age and to the MRC total score and we observed a bell distribution both in DMD and in BMD patients (Figures 2E,F). Younger and stronger patients had low levels of PDGF-AA; patients in an intermediate stage that were losing muscle strength had higher levels of PDGF-AA, while patients in the end-stage of the disease, that were very weak or older, had again low levels of PDGF-AA. Muscle strength measured by MRC significantly correlated with TGF- β1 levels in DMD patients (*p* = 0.026, Spearman's rho = 0.523) and with CTGF levels in BMD patients (*p* = 0.012, Spearman's rho = −0.669). We did not find statistically significant correlations between the PDGF-BB serum levels and the function tests.

**Table 2:** Correlation between PDGF-AA serum levels and different muscle function tests, spirometry values, and muscle MRI.

	**DMD**		**BMD**	
**Test**	**Correlation coefficient**	**Spearman *p*-value**	**Correlation coefficient**	**Spearman *p*-value**
10MWT	0.738	0.037	0.9	0.001
6MWT	−0.637	0.026	−0.929	0.001
Tup4	0.287	0.365	0.9	0.001
Tdo4	0.105	0.746	−0.867	0.012
TRF	0.406	0.191	0.964	0.001
MRCT	−0.537	0.022	−0.398	0.178
Pinch	0.574	0.082	0.452	0.260
Grip	0.676	0.152	0.476	0.233
PUL	0.741	0.205	0.467	0.302
MFM-D3	0.975	0.005	−0.61	0.885
Egen klassification	−0.700	0.188	0.300	0.625
Activlim	0.521	0.231	−0.287	0.490
NSAA	−0.124	0.717	−0.952	0.001
FVCs%	0.500	0.391	0.738	0.037
FVCl%	0.400	0.505	0.524	0.183
FEV1%	0.800	0.104	0.810	0.015
MRI Thigh FF	−0.179	0.702	−0.786	0.021

*Significance was analyzed using Spearman test. 10MWT, 10 meters run/walk test; 6MWT, 6 minutes walking test; Tup4, Time to climb up 4 steps; Tdo4, Time to go down 4 steps; TRF, Time to rise from floor; MRCT, Composite score of the muscle strength calculated using MRC score in both upper and lower limbs; PUL, Performance of the Upper Limb scale; MFM-D3, Motor Function Score 20 items (distal dimension); Activlim, ACTIVity LIMitations scale; NSAA, North Start Ambulatory Assessment; FVCs%, Force Vital Capacity Percentage Predicted while sitting; FVCl%, Force Vital Capacity Percentage Predicted while lying; FEV1%, Forced expiratory volume in the first second; MRI Thigh FF, fat fraction in thighs*.

### PDGF-AA Expression in Skeletal Muscle Biopsies of DMD and BMD Patients

**Figure 3:**
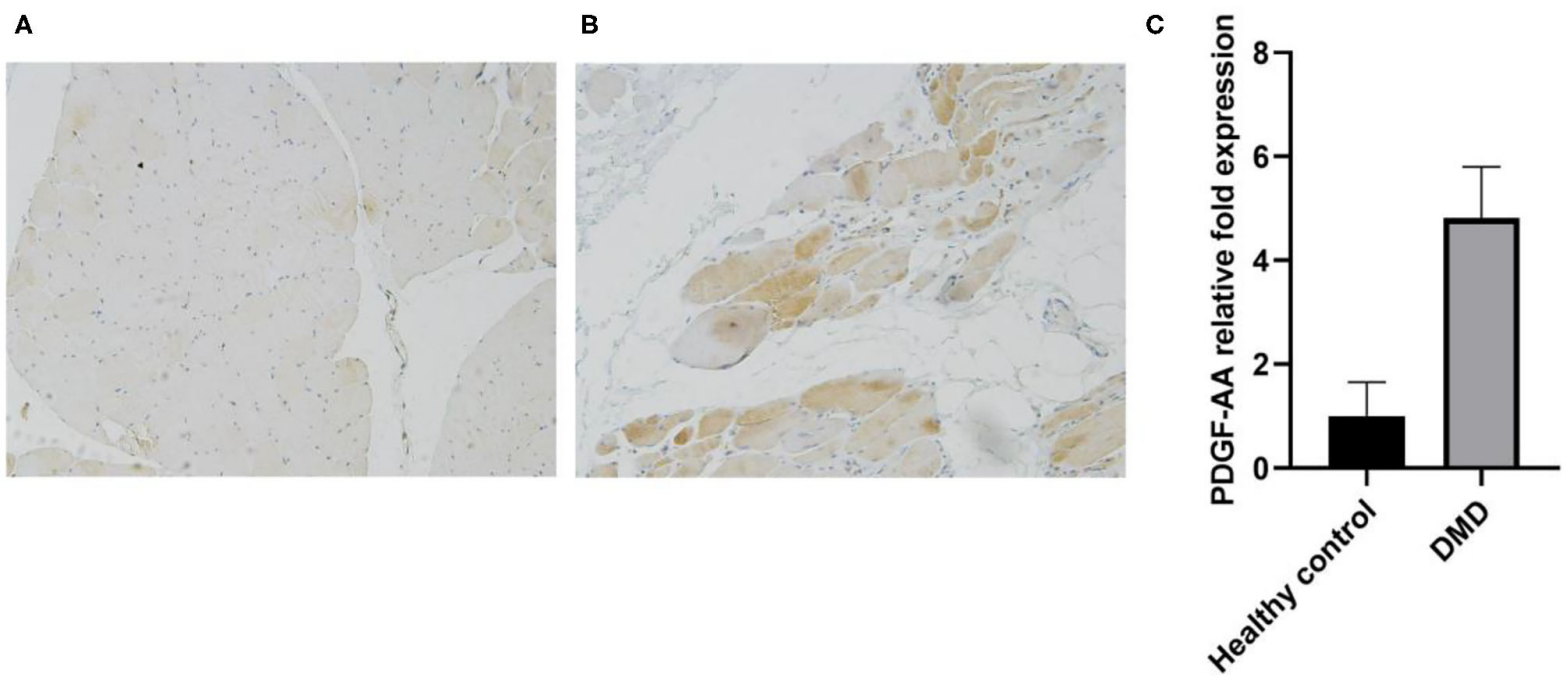
PDGF-AA expression is increased in muscles of patients with dystrophinopathy. **(A)** PDGF-AA expression in skeletal muscles of healthy controls; **(B)** PDGF-AA expression in a muscle biopsy of a DMD patient and **(C)** Real Time PCR showing PDGF-AA mRNA expression in skeletal muscle samples of controls and patients with dystrophinopathy. Bars show mean mRNA expression and standard deviation.

In a final step we studied PDGF-AA expression patterns in muscle biopsies of patients with DMD and BMD and in controls using immunohistochemistry and Real Time PCR. As it has been previously shown, PDGF-AA is expressed in the capillaries in muscles of healthy controls, where it probably has a role in binding the pericytes to the endothelial cell (Figure 3A) (22, 23). In patients with DMD, we observed that muscle fibers expressed PDGF-AA as it is shown in Figure 3B. qPCR confirmed the results of immunohistochemistry and showed an increased expression of PDGF-AA in muscles from DMD compared to healthy controls (Figure 3C).

## Discussion

The goal of the work behind this manuscript was to identify serum candidate biomarkers to track progression in DMD and BMD.

The current development of new therapies for muscle dystrophies, partly due to the progress in the field of genetics in the latest decades, raises the need for reliable outcome measures. DMD and BMD are slow progressive diseases where weakness is developed over years. However, clinical trials designed for these disorders must be able to prove presence or absence of effect in a shorter period of time. Biomarkers that can closely track the pathophysiological mechanisms of these diseases, and therefore be used as outcome measures, would be desirable. The research in the field has been centered in two different measurements. On the one hand, qMRI has demonstrated to be a reliable tool to analyze changes in muscle structure in patients with muscular diseases. Fat replacement, assessed using Dixon imaging or spectroscopy could be a suitable outcome measure, because it correlates with muscle function and it is sensitive to changes over short periods in several disorders such as DMD, Pompe or LGMD-2I (7, 15, 24). On the other hand, blood biomarkers are especially interesting because they are easy to obtain. Serum concentration of molecules such as cytokines, metalloproteinases or microRNAs have shown promising results in some neuromuscular diseases (16, 25). Creatine kinase (CK) is a widely accepted biomarker for diagnosis of DMD, BMD and other muscular dystrophies. However, its levels fluctuate and are influenced by different conditions such as physical activity, therefore it is not considered a good biomarker to study diseases' progression (16). Other molecules present in muscle and involved in the pathophysiology of muscular dystrophies have been studied. For example, Hathout et al. (26) found 44 proteins which were able to differentiate DMD patients from healthy volunteers. Other studies aimed not only to differentiate between patients and controls, but also to correlate with functional state and treatment (27–29) or muscle function tests (30). Fewer manuscripts aim to provide longitudinal data on the suggested biomarkers (31–33).

We were interested in investigating the serum concentration of PDGF-AA, PDGF-BB, CTGF, and TGF β1 in our patients because of their role in fibrosis. The process of muscle fibrosis in patients with muscular dystrophies is highly complex and not completely understood (34). It is known that chronic muscle degeneration leads to persistent inflammatory infiltration, muscle necrosis and activation of fibroadipogenic progenitor cells (FAPs) and fibroblasts, which release proteins of the extracellular matrix such as collagen-I, leading to the expansion of fibrotic tissue (34–36). The PDGF family in general and PDGF-AA in particular play an important role in this process (22, 37). PDGF-AA is produced by different cell types, such as platelets, muscle fibers or macrophages and acts via the PDGF receptor alpha (PDGFRα) (38). When PDGF-AA binds to its receptor, several signaling pathways involved with cellular proliferation, differentiation and motility are triggered (39). After an acute muscle injury, damaged fibers and inflammatory cells release several cytokines, including PDGF-AA, which activate FAPs inducing the release of extracellular matrix components (40). Extracellular matrix is needed for muscle regeneration as it acts as a scaffold for muscle fiber regeneration (41). When muscle regeneration is completed, inflammatory cells and the new muscle fibers release TNF-α that promotes apoptosis of FAPs and fibroblasts reducing their number and avoiding the development of fibrosis. At that stage, levels of PDGF-AA decrease (42). However, in dystrophinopathies where there is chronic muscle damage, inflammatory cells permanently infiltrate the injured muscle continuously releasing PDGF-AA, which expand the number of activated FAPs leading to an increased non-controlled fibrotic response (40, 43). Consequently, treatment of murine models of DMD with tyrosine kinase inhibitors that block the PDGFRα such as imatinib, crenolanib and nintedanib, decreased fibrosis and improved muscle function of these animals (40, 42, 44). We think that due to this increased pro-fibrotic response in patients with DMD, the levels of PDGF-AA are high in comparison to controls, as shown in our study.

Other primary fibrotic growth factors including the TGF-β1 family and CTGF have also been reported to be involved in the process of muscle fibrosis (36, 45, 46). TGF-β1 and PDGF-BB participate in mesenchymal cell proliferation and fibrotic remodeling (47, 48) and CTGF stimulates chemotaxis and proliferation of fibroblasts and the production of extracellular matrix components (45).

Despite its role in the physiopathology of muscle degeneration, there are only few reports studying the potential utility of growth factors as biomarkers of the disease (49–51).

In our study, the serum concentration of PDGF-AA was increased in patients with DMD in comparison to pediatric controls. There was also an increase in PDGF-AA levels in BMD patients compared to adult controls, although it did not reach statistical significance. Moreover, PDGF-AA serum levels correlated with several muscle function tests both in DMD and BMD, and with the results of Dixon MRI in BMD. Because of the common underlying pathophysiologic mechanism of both DMD and BMD, we speculate that serum levels of PDGF-AA should be further studied in a larger cohort of DMD/BMD patients and considered as a potential biomarker for the disease.

Our results suggest that the expression of PDGF-AA in DMD and BMD patients follows a bell curve distribution. In initial stages of the disease, when histological changes are consistent with acute muscle damage, PDGF-AA expression by muscle fibers is still low, similarly to that observed in controls. As the disease progresses, muscle fibers degenerate leading to increased production of PDGF-AA that potentially activates FAPs involved in the expansion of fibrotic and adipogenic tissue (22). In advanced stages, when most of the muscle has been replaced by fibrotic and fatty tissue, levels drop again as there are no more muscle fibers releasing PDGF-AA. This hypothesis allows us to understand the results obtained in the correlations between PDGF-AA levels and the muscular function of the patients. Patients in early stages of the disease, in whom muscle strength assessed using the MRC scale is only mildly affected, have low levels of PDGF-AA. These patients in early stages are still able to walk, have high scores on the NSAA, need little time to walk 10 m and cover long distances in the 6MWT. Patients in an intermediate stage of the disease have high levels of PDGF-AA and are clearly weak as it is demonstrated using the MRC scales. These patients are able to cover 200–400 m in the 6MWT, but they still have intact respiratory muscle strength. In contrast, patients in advanced stages of the disease have lower levels of serum PDGF-AA, are extremely weak as assessed with the MRC scale and have higher fat replacement in the muscles. Moreover, these patients are not able to walk and have affected respiratory muscles as shown using the spirometry. Although levels of CTGF were also increased in BMD patients in comparison to controls, we only found significant correlations with the MRC scale.

The process of fibrosis is not exclusive to muscular tissue. In fact, fibrotic tissue remodeling can affect virtually every system. Both primary fibrotic diseases such as systemic sclerosis and idiopathic pulmonary fibrosis and secondary fibrotic responses in other organs share many commonalities. An initial injury triggers the reparative process in which lymphocytes and macrophages release profibrotic mediators. These mediators promote the activation of myofibroblasts which liberate extracellular matrix proteins leading to structural changes. Therefore, it is not surprising to find that the growth factors that we have studied here are also implicated in other diseases such as atherosclerosis, liver and pulmonary fibrosis, cirrhosis, myocardial infarction and neoplasms (52–58). However, the primary site of injury, the cells that differentiate into myofibroblasts and the organization of the immune response differs among the different types of disorders (52). Interestingly, in our study TGF-β1 showed a different behavior in BMD than in DYSF patients, since TGF-β1 levels were increased in BMD patients compared to DYSF patients. In our opinion this data suggests that, although the final stage of the disease may be common among muscular dystrophies, the process leading to replacement of muscle fibers by fibrotic and fatty tissue may have some particularities that could be exclusive to each type of muscular dystrophy, such as the type of muscle injury or the molecular pathways that are activated in response to this muscle injury. However, we did not find significant differences in TGF- β1 between patients and controls (only between BMD and DYSF patients) and therefore these results should be taken with caution.

The four growth factors that we have studied have been previously related with fibrosis and with muscle regeneration in muscular dystrophies (59, 60) but have also been studied in other neuromuscular diseases. For instance, our group identified a decrease in PDGF-BB levels in patients with Pompe disease suggesting that there is a deficit in muscle regeneration in this disease, and Sato et al. showed that CTGF levels were high in patients with polymyositis and dermatomyositis (61, 62).

This is a small study with several limitations: firstly, it is a transversal study without longitudinal data. Secondly, only half of the patients underwent Dixon MRI, and the ones who did were already severely affected by the disease and therefore had high FF.

Despite these limitations, our study suggests that serum levels of PDGF-AA can differentiate between DMD patients and controls, and that they correlate with the results of different muscle tests. For BMD patients we have also found correlation between PDGF-AA levels and muscle function tests, including fat muscle content assessed by Dixon MRI. Based on these results, we propose PDGF-AA as a growth factor to be further investigated in in a larger cohort of patients and in longitudinal studies.

## Data Availability Statement

The original contributions presented in the study are included in the article/supplementary material, further inquiries can be directed to the corresponding author/s.

## Ethics Statement

The studies involving human participants were reviewed and approved by Hospital de la Santa Creu i Sant Pau Ethics Committee and Hospital de Sant Joan de Deu Ethics Committee. Written informed consent to participate in this study was provided by the participants' legal guardian/next of kin.

## Author Contributions

AA-J, EF-S, and JD-M contributed to the study conception and design. Clinical evaluation of patients was performed by AA-J, JD-M, JA-P, DN-d, CO, and CJ-M. Blood samples and data collection was obtained by SS. EG, JD-M, and EF-S designed and performed the experiments. PP-J, AC-R, and XS-C also collaborated with the execution of the experiments. PM, JD-M, CN-P, and JL designed the MRI protocol. The analysis of the MRI images was performed by AA-J. Functional assessments were performed by IB, IP, CG, and EM. AA-J, EF-S, and JD-M analyzed the results and wrote the manuscript. All authors contributed with their comments to the final version.

## Conflict of Interest

PM is employed by the company Philips Healthcare Iberia. The remaining authors declare that the research was conducted in the absence of any commercial or financial relationships that could be construed as a potential conflict of interest.
